# Validity of Weight Estimation Models in Pigs Reared under Different Management Conditions

**DOI:** 10.1155/2014/530469

**Published:** 2014-05-28

**Authors:** Marvelous Sungirai, Lawrence Masaka, Tonderai Maxwell Benhura

**Affiliations:** Department of Livestock and Wildlife Management, Midlands State University, Private Bag 9055, Gweru, Zimbabwe

## Abstract

A study was carried out to determine the relationship between linear body measurements and live weight in Landrace and Large White pigs reared under different management conditions in Zimbabwe. Data was collected for body length, heart girth, and live weight in 358 pigs reared under intensive commercial conditions. The stepwise multiple linear regression method was done to develop a model using a random selection of 202 records of pigs. The model showed that age, body length, and heart girth were useful predictors of live weight in these pigs with significantly high positive correlations observed. The model was internally validated using records of the remaining 156 pigs and there was a significantly high positive correlation between the actual and predicted weights. The model was then externally validated using 40 market age pigs reared under communal conditions and there was a significantly low positive correlation between the actual and predicted weights. The results of the study show that while linear measurements can be useful in predicting pig weights the appropriateness of the model is also influenced by the management of the pigs. Models can only be applicable to pigs reared under similar conditions of management.

## 1. Introduction

In pig production it is always important to know the weight of pigs at a given time. Such knowledge is vital for a number of reasons which include determination of feed requirements [1], animal health status, determination of growth rates, determination of time when animals are sent to market, space allowances, and determination of drug dosages [2]. Accuracy of predicting pig weight leads to profitability in commercial farms due to the reduction in feed costs which account for 60% of production as feed requirements are accurately calculated [3]. Costs are also reduced in the treatment of diseases as there is no overestimation of weights and underestimation of weight could be potentially dangerous due to the development of drug resistance. According to Zaragoza [3], there are basically two main approaches which could be used to estimate the weight of pigs; these are the direct and indirect approaches. The direct method involves physically moving the pigs to a weighing location and placing them on a weighing scale. Several authors [4–7] have described the disadvantages of using the direct methods and these include requirements for high input of labor, changes in the feed behavior of pigs which might lead to weight loss, stress which at times can lead to death, and injury occurring to the people working with the pigs. In addition to that, the weighing scale may become inaccurate due to the constant physical contact of the machine with the animal and the dirty environment [2]. On the other hand, the indirect method involves visual estimation of weight, the use of linear body measurements, and image analysis [3]. Of the indirect methods, the use of linear body measurements is the most common tool that is used to predict body weight in farm animals. The heart girth, body length, height at withers, and flank-flank measurements are the major measurements used in weight estimation.

In Zimbabwe, there is a paucity of published information which seeks to describe the relationship between linear body measurements and weight in pigs of different breed, sex, and age. Although several studies [3–11] have described the relationship between the linear body measurements and pigs in other countries, there is no published work in Zimbabwe which particularly looks at how these could be different in animals of different breeds, sex, and age. Furthermore an argument is put forward by [7] that such relationships could differ in different environments. The aim of this study was to determine the influence of fixed factors (age, breed, and sex) on the relationship between the linear body measurements (heart girth and body length) at a commercial pig farm and to find out if the established relationship could be extrapolated to pigs reared under different management conditions.

## 2. Materials and Methods

### 2.1. Study Site

The study was carried out at Lisheen Estate which is located 30 km east of Harare. It is an intensive farming area, specializing in livestock and crop production.

### 2.2. Experimental Animals and Management

Three hundred and sixty pigs of Landrace (*n* = 180) and Large White breeds (*n* = 180) from the farm were divided into different categories of breed, sex, and age which were regarded as the fixed factors. The management of the pigs in all the categories had been the same. At birth, the piglets were given colostrum and their navels were dipped with iodine solution. The eye teeth were clipped on the second day to prevent piglets from inflicting wounds on the teats of the sow. The piglets were also injected with a solution of ferrum (iron dextran) to supplement for iron. The animals were ear notched for identification purposes. The piglets were given creep feed* ad lib* (21% CP) for eight weeks and after they had reached a weight of 20 kg they were weaned. After that, pigs were* ad libitum* fed diets suitable for the growing and fattening period (16% CP). Breeding animals received a less concentrated feed (13% CP) at the rate of 2 kg per animal per day. The animals were given preventive doses of ivermectin to protect against internal parasites. A further 40 pigs sourced from neighbouring small holder farms which had reached market age were used in model validation.

### 2.3. Data Collection

Body length and heart girth were measured using a clothing tape after the animal had been restrained with a hog strainer. Body length was defined as the length from the base of the neck to the base of the tail [7] and heart girth was defined as the circumference of the chest area just behind the forelegs where the tape was placed directly behind the front legs and then wrapped around the heart girth and read directly behind the shoulders [6]. Two spring scales were used to weigh the pigs, one for smaller animals and the other for larger animals. To improve accuracy, the small pigs were placed in sacks and suspended from the scale and the weights were recorded while the larger pigs were suspended by means of ropes [12]. The information was collected on data sheets and then entered into the Microsoft Excel spreadsheet.

### 2.4. Data Analysis

The data entered into the Microsoft Excel spreadsheet was cleaned and checked for errors and inconsistencies in data collection and records of 358 pigs were then used for data analysis. Statistical analysis was performed using the SPSS (Statistical Package for Social Sciences) version 16 software. The stepwise regression method was done to determine the independent variable which was a good estimator of weight in Large White and Landrace pigs of different sexes. The goodness of fit (*R* ^2^) was used to determine the contribution of the variables to the prediction of body weight and the *P* values from the regression analysis of variance were used to find out if the contributions were significant or not. The accuracy of the equation was estimated using residuals which is the absolute value of the difference between predicted weight by using the developed equations and actual weight measured with the scale [1].

### 2.5. Model Validation

The model was validated using two procedures. Internal validation was done using the cross-validation method, where 202 pigs were used to create the model and the remaining 156 pigs validated the model. The procedure was repeated with the 156 pigs creating the second model and the 202 pigs validating the second model. External validation was done using 40 pigs from a different population using the model from internal validation which had been found to be the best predictor of live weight.

## 3. Results

**Figure 1:**
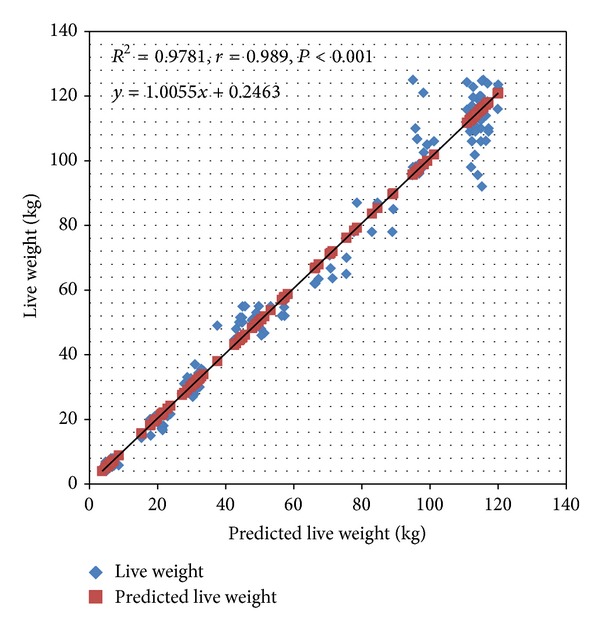
Cross-validation of model 1 (*n* = 202) with the second group of pigs (*n* = 156).

Two hundred and two pigs were used to come up with a prediction model (model 1) and the correlations of the variables are shown in Table 1. As can be seen all the correlations except for breed and sex were statistically significant. The prediction model contained three of the five predictors and was reached in three steps with two variables removed (breed and sex). The model was statistically significant, *F* (3, 198) = 2 283, *P* < 0.001, and accounted for about 97% of the variation in live weight (*R* ^2^= 0.972, adjusted *R* ^2^ = 0.971, see Table 2). Live weight could be predicted strongly by the age of the animal followed by the body length and the heart girth (*r* = 0.986). The prediction equations for each of age, body length, and heart girth are shown in Table 3. The prediction model developed from the 202 pigs was used to estimate the weights of the 156 pigs and the results of the estimates were correlated with the actual weights of the 156 pigs and a strong correlation was observed between the predicted and actual weights (*r* = 0.989, *R* ^2^ = 0.978 and the correlation was significant, *F* (1, 154) = 6 885, *P* < 0.001); see Figure 1.

**Figure 2:**
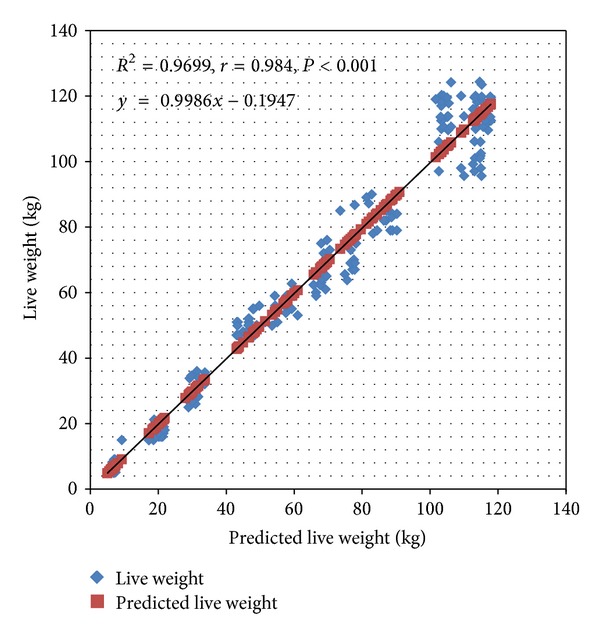
Cross-validation of model 2 (*n* = 156) with the first group of pigs (*n* = 202).

A prediction model was also developed (for cross-validation) using the 156 pigs (model 2) and this model also removed breed and sex and retained age, body length, and heart girth measurements in that order which was the same for the model produced with the 202 pigs (see Table 4). The prediction model developed from 156 pigs was used to estimate the weights of 202 pigs and the results of the estimates were correlated with the actual weights of the 202 pigs and a strong correlation was observed (*r* = 0.984, *R* ^2^ = 0.967) and it was statistically significant (*F* (1, 201) = 5 956, *P* < 0.001); see Figure 2. In this case, the correlation and the percentage of variation accounted for were slightly less than when the 202 pigs had been used to predict the model and validated with the 156 pigs. Therefore model 1 was used for further analysis.

**Figure 3:**
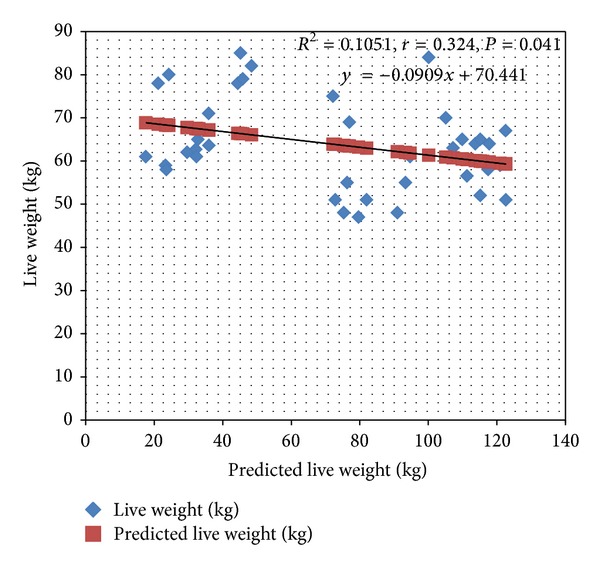
External validation of model 1 with pigs from differently managed environment (*n* = 40).

Model 1 was further subjected to external validation using the 40 pigs drawn from neighboring farms and managed differently from the pigs used to develop model 1. Their breeds could not be ascertained but were suspected to be crossed between Large White and Landrace breeds. Their actual weights were correlated with the predicted weights using body length only, heart girth only, and a combination of both length and girth. The results in Table 5 show that the prediction model was a poor estimator of weight in pigs not drawn from the same farm as the one used to come up with model 1; this is shown by the low correlation values of less than 0.5 and low values of the percentage of variances (*R* ^2^) that account for the relationship observed; see also Figure 3.

## 4. Discussion

In this study, breed and sex did not influence the estimation of live weight in pigs but age of the animal, heart girth, and body length did. This is in agreement with Benyi [13] who found out that the breed or sex of goats did not have any influence in the estimation of live weight in goats; this is usually the case when the animals are well managed in a uniform environment free of stress, poor nutrition, health, and management. Though Benyi's study was done in goats a similar explanation could be given in this case since the pigs used in this study were reared in the same environment and as such breed and sex did not have any effect on the estimation of live weight in the animals and furthermore it was attempted to keep the effects of stress on the animals at a minimum. The study showed that there was a high positive correlation between linear body measurements and live weight in Large White and Landrace breeds. This is in line with the study carried out by Machebe and Ezekwe [7] who reported a correlation coefficient of 0.97 for body length as well as 0.98 for heart girth. In this study body length explained approximately 98% of the variation in the relationship between body length and live weight in pigs whilst heart girth explained 89% of the variation on the same relationship. Similar values have been reported by other authors [3, 6, 12]. The same results have also been observed in goats and cattle species [1, 13–15]. However in this study it was shown that body length contributed more to the variation compared to heart girth whereas in previous studies it has been concluded that heart girth gives the best estimate of live weight not only in pigs but in other species as well. This could be explained by the fact that a lot could go wrong when taking linear body measurements, for instance, pigs move around and have a tendency to lift their heads [7], hence affecting the accuracy of the results. In the present study, the use of a hog restrainer probably reduced the ease of measurement of heart girth resulting in a lower correlation with weight compared to that reported in literature.

Another important finding in this study was that age could be used in the estimation of live weight as it showed a high correlation and also explained more of the variation in the estimation of live weight compared to other predictors, that is, body length and heart girth. Mutua and colleagues [12] have proposed the use of an age-specific model in the development of weight estimation charts in pigs. Furthermore Brandl and J*ø*rgensen [2] have described age as one of the factors that would influence weight estimation in pigs. Kunene et al. [16] also found that age did significantly influence linear body measurements in sheep. Looking at the differences in the percentage of variation accounted for by each of the predictors age, body length, and heart girth, it is seen that each of them can estimate live weight equally accurately.

In this study, the model developed could not accurately estimate the weights of pigs drawn from neighboring farms but could accurately predict the weights of pigs raised on the same farm. Nwosu et al., 1985, cited in [7] established that weight estimation in cattle differed between two environments. This has largely to do with the management style employed in each of the operations. In this case, it is suggested that the management style of the 40 pigs brought to the farm was different from the one practiced at the farm. The 40 pigs were largely drawn from small scale pig farmers whilst the 202 pigs used to develop the model where drawn from an intensive farming operation. The pigs from the small scale farmers were largely crossbreeds of Landrace and Large White, given the management differences between the two sets of pigs; it is conceivable that breed differences as well as sex differences could become manifested. Differences in nutrition management can also result in the impossibility of extrapolating models as the growth patterns of pigs respond to changes in planes of nutrition. Small scale farmers tend to supplement concentrate feeds with crop residues and feed wastes from the household which are likely going to be of lower nutritional value. Therefore due to these combinations of factors affecting the growth characteristics of pigs, by the time the pigs from small scale communal farmers reach market age their weight is not comparable to that of pigs reared under intensive management conditions and this will subsequently render weight estimation models to be applicable only to animals that are reared under similar management conditions.

## 5. Conclusion

Weight estimation models using linear body measurements are tailor made for a particular population of pigs. While they provide a viable alternative for both large scale and small scale farmers they are more suitable for a commercial setup as pig management is more tightly controlled compared to the latter. Another factor that constrains the applicability of weight estimation models in small scale farming is the lack of proper record keeping. As seen in the present study breed and sex differences could become manifested under different management conditions and small scale farmers are usually unaware of the breeds that they keep.

## Figures and Tables

**Table 1:** Pearson's correlation coefficient between live weight and the independent variables (*n* = 202).

Variable	Body length	Heart girth	Age	Breed	Sex	Live weight
Body length	—	—	—	—	—	—
Heart girth	0.933	—	—	—	—	—
Age	0.980	0.942	—	—	—	—
Breed	0.021	0.002	0.008	—	—	—
Sex	−0.163	−0.170	−0.191	0.02	—	—
Live weight	0.977	0.944	0.980	0.028	−0.192	—

**Table 2:** Stepwise multiple linear regression prediction of live weight from body measurements (*n* = 202).

Age	Body length	Heart girth	Intercept	*r*	*R* ^2^
2.910			−3.218	0.980	0.960
1.655	0.526		−20.999	0.985	0.970
1.409	0.477	0.283	−26.643	0.986	0.972

**Table 3:** Relationship between linear body measurements and live weight in Landrace and Large White pigs (*n* = 202).

Component	Prediction equation	*r*	*R* ^2^
Age	Live weight = −3.218 + 2.91 age	0.980	0.960
Body length	Live weight = −41.157 + 1.184 body length	0.977	0.954
Heart girth	Live weight = −50.153 + 2.067 heart girth	0.944	0.892

**Table 4:** Stepwise multiple linear regression prediction of live weight from body measurements (*n* = 156).

Age	Body length	Heart girth	Intercept	*r*	*R* ^2^
2.923			−3.501	0.988	0.976
2.195	0.314		−14.209	0.989	0.978
2.02	0.254	0.232	−17.777	0.990	0.979

**Table 5:** Correlation between prediction model and 40 pigs from neighboring farms.

Model used for correlation	*r*	*R* ^2^	Significance at *σ* = 0.05
Length only	0.347	0.12	*P* = 0.028
Heart girth only	0.241	0.058	*P* = 1.33
Heart girth + length	0.324	0.105	*P* = 0.041
